# Social Isolation: A Narrative Review on the Dangerous Liaison between the Autonomic Nervous System and Inflammation

**DOI:** 10.3390/life13061229

**Published:** 2023-05-23

**Authors:** Costanza Scatà, Angelica Carandina, Alice Della Torre, Beatrice Arosio, Chiara Bellocchi, Gabriel Dias Rodrigues, Ludovico Furlan, Eleonora Tobaldini, Nicola Montano

**Affiliations:** 1 Department of Internal Medicine, Fondazione IRCCS Ca’ Granda Ospedale Maggiore Policlinico, 20122 Milan, Italy; 2 Department of Clinical Sciences and Community Health, University of Milan, 20122 Milan, Italy

**Keywords:** social isolation, cardiovascular autonomic nervous system, immune system

## Abstract

Social isolation and feelings of loneliness are related to higher mortality and morbidity. Evidence from studies conducted during space missions, in space analogs, and during the COVID-19 pandemic underline the possible role of the autonomic nervous system in mediating this relation. Indeed, the activation of the sympathetic branch of the autonomic nervous system enhances the cardiovascular response and activates the transcription of pro-inflammatory genes, which leads to a stimulation of inflammatory activation. This response is adaptive in the short term, in that it allows one to cope with a situation perceived as a threat, but in the long term it has detrimental effects on mental and physical health, leading to mood deflection and an increased risk of cardiovascular disease, as well as imbalances in immune system activation. The aim of this narrative review is to present the contributions from space studies and insights from the lockdown period on the relationship between social isolation and autonomic nervous system activation, focusing on cardiovascular impairment and immune imbalance. Knowing the pathophysiological mechanisms underlying this relationship is important as it enables us to structure effective countermeasures for the new challenges that lie ahead: the lengthening of space missions and Mars exploration, the specter of future pandemics, and the aging of the population.

## 1. Introduction

The need for closeness to others lingers throughout the lifetime of an individual [1]. From an evolutionary perspective, social isolation represents a risk factor for individual survival, exposing the person to the danger of attacks from animals or other rival conspecifics. Therefore, it is not surprising that the physical pain system can be co-opted by social pain. This dual activation operates as an internal alarm, causing distress if we move away from those we love and warning us of potential or actual damage [1,2].

Social isolation and feelings of loneliness are correlated with global worse health status: those who are less embedded within a valid social network are more likely to become sick, and to recover more slowly from illness and from surgery [3]. Several studies have shown an important correlation between social isolation and higher mortality risk [4,5,6]. A meta-analysis by Lunstad and colleagues estimated that social isolation, loneliness, and living alone increased the possibility of death by 29%, 26%, and 32%, respectively, representing a condition comparable with well-established risk factors for mortality, such as physical inactivity, obesity, or substance abuse [7]. However, the pathophysiological mechanisms underlying this dangerous relationship have yet to be elucidated.

Settings such as space missions, space analogs, and even the restrictions imposed to contain the spread of the virus during the COVID-19 pandemic are interesting contexts in which to study the psychosomatic consequences of social isolation. In particular, it has been observed that social isolation can be associated with increased cardiovascular morbidity and mortality [8,9], as well as impairment in the immune system and reduction in the resistance to diseases and infections [3,10]. 

Because of its pervasiveness and its negative effects, it is important to consider social isolation as a relevant public health problem and to identify which pathophysiological mechanisms are involved in order to improve effective countermeasures and enable data sharing between spatial and terrestrial environments [11]. Thus, in the present narrative review, moving from the evidence observed in controlled environments such as those of space missions and space analogs and from the experience of COVID-related lockdowns, we want to propose an overview of the stress response triggered by social isolation and the pathophysiological alterations that derive from time-prolonged recruitment of this stress response. Furthermore, we propose the autonomic nervous system (ANS) as a liaison between social isolation, feelings of loneliness, and the psychosomatic symptoms observed in these study models.

## 2. Social Isolation and Stress Response

Social isolation is one of the most important stress factors to consider in space missions and is among the main categories listed by NASA as risk factors for the success of spaceflight. The delays in communication with Earth, the distance from loved ones, and the cultural and language differences can be particularly difficult to manage and can generate a feeling of loneliness and isolation, with detrimental effects at individual and group level [12,13,14]. At present, interest in space missions is increasing and technological knowledge has enabled the lengthening of the missions themselves. Thus, the issue of social isolation becomes a matter of great importance, to be addressed both in terms of its negative effects on the physical [15] and mental [16] health of the individual, and in terms of personal adaptation and group collaboration [17,18] and its consequences, including increased cardiovascular risk [19], dysregulation of the human immune system [20], and impaired cognitive and mood performance [21]. 

The need to improve knowledge on the pathophysiological mechanisms activated by social isolation that lead to an impaired health status in astronauts and the possibility of translating these findings to larger populations affected by social isolation increases the interest in controlled environments such as those of spaceflights and space analogs. Space analogs, such as purpose-built simulated space capsules or polar stations like the Antarctic Concordia Station, are isolated and confined extreme (ICE) environments in which it is possible to study more easily and cheaply the psychological and physical stressors that affect astronauts during space missions [22,23]. In these settings, the detrimental effects of confinement and social isolation are assessed on healthy individuals in a controlled way, to identify the pathophysiological pathways involved and to study possible countermeasures. It has been observed that disturbed sleep, impaired cognitive abilities, and mood deflection frequently occur among people who have participated in missions in space analogs [23,24], as observed in the 60% of cases in Antarctica missions [25,26]. The evidence obtained in these controlled environments has the possibility of being extended to the general population, for example to protect the health status of frail populations who often live in situations of loneliness and isolation (e.g., the elderly, psychiatric patients). 

Another important study setting is related to the COVID-19 pandemic, during which almost all countries imposed social distancing strategies and lockdowns to prevent the spread of the virus. Therefore, the pandemic can be seen as the largest experiment on social isolation in history, and this makes a unique contribution to understanding the effects of a long period of social isolation on healthy individuals. The prevalence of loneliness following COVID-19 restrictions was rated to vary between 16 and 25%, and was probably underestimated [27,28]. Lockdown proved to be linked to increased distress, depression, anxiety, and posttraumatic stress symptoms, as well as perceived cognitive impairment, especially among young women [29,30,31,32]. Studies on the COVID-19 pandemic are useful to investigate the transition from a state of health to the onset of psychosomatic symptoms related to prolonged social isolation, as well as to understand the negative consequences of social isolation on brain and behavior in order to improve the comprehension of how to mitigate related adverse conditions. 

From an evolutionary perspective, being alone means being more susceptible to encounters with predators or other groups of enemies, and this condition of increased personal risk triggers a stress response to cope with the threat. According to Selye’s theory [33], any potentially life-threatening agent, regardless of its nature, activates in the body a non-specific stress response termed “General Adaptation Syndrome”. 

Selye divided the response into three distinct stages. At first, this activation allows the body to counteract the threat, but if sustained the body exhausts its adaptive capacity. During the first phase, the “alarm reaction stage”, the body prepares itself to cope with the stressor by activating two main physiological systems: the hypothalamus–pituitary–adrenal (HPA) axis, and the sympathetic branch of the ANS (SNS). The latter system allows the body to perform the “fight or flight response”, involving the release of catecholamines, breath acceleration, the activation of the inflammatory response, and an increase in heart rate and blood pressure [33]. In contrast, the neurohormonal correlate is characterized by the activation of the HPA axis, resulting in corticotropin release and increased SNS activation [34]. After this initial massive activation, the body needs to return to pre-stress levels, with the normalization of the cardiovascular functions and a reduction in cortisol and adrenaline/noradrenaline production [35]. This second phase, named by Selye as the stage of “resistance”, is particularly important as the body remains in a state of alertness. If it has effectively counteracted the threat, the body returns to the pre-stress baseline situation, but if the stressor is still present, it eventually adapts to a higher-than-usual level of activation to cope with the threat. A situation of chronic or prolonged stress generates an exaggerated physiological and hormonal activation that weakens the body and can lead to severe complications [33]. This is the moment in which the response activated to cope with the danger becomes dangerous itself, leading to the “exhaustion” phase, in which the body no longer has physical or mental resources to counteract the situation of chronic stress, leading to burn-out and to an increased risk of developing stress-related illnesses [36]. Notably, the ANS responds immediately through the activation of the SNS, whereas the HPA axis has a major role in the second phase through the maintenance and down-regulation of the response itself [37]. The prolonged activation of these two systems generates the damaging effects of the last phase. In particular, the sustained activation of the SNS, without an adequate counteraction of the parasympathetic nervous system (PNS), leads to increased cardiovascular morbidity and mortality [38] and increased pro-inflammatory cytokines production, resulting in immune dysregulation [39]. Thus, the inhibition of the SNS or the enhancement of the PNS could help to mitigate adverse effects and to interrupt the cycle before a down-regulation is no longer possible.

Social isolation has direct effects on the neurovegetative response and can trigger “fight or flight”-type defense responses, involving both the HPA axis and the increase in SNS tone [4,40,41]. The disruption of social boundaries activates a stress response originating in the central nervous system (CNS) that prepares the body to cope with the threat by raising the level of cortisol [42,43] and by the activation of the sympathetic branch of the ANS [8,42]. 

**Figure 1:**
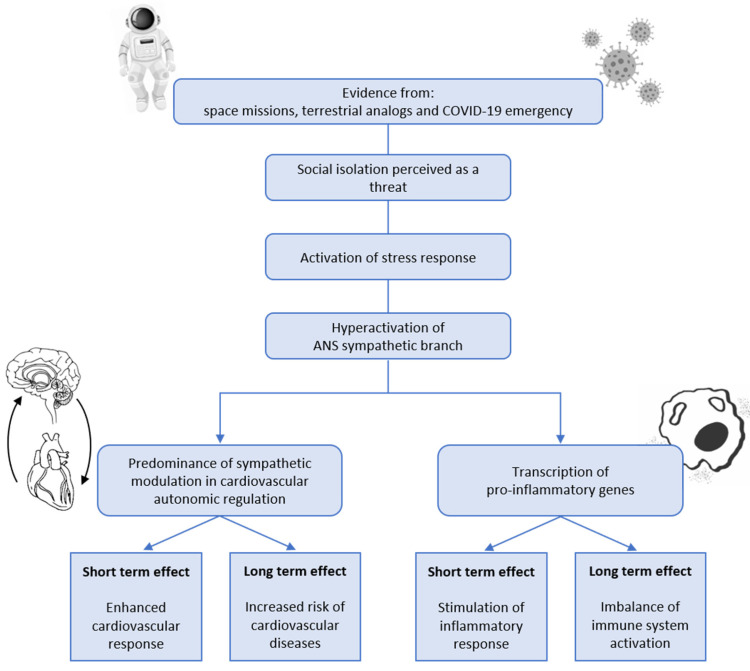
Pathophysiological link between stress response and alterations induced by social isolation and the different settings in which this has been studied.

Several studies on the pathophysiological mechanisms of social isolation have been performed on both humans and animal models, and the latter are particularly useful because it is possible to experimentally manipulate social isolation in order to observe both its acute and chronic effects on HPA and SNS activation [4]. In general, when animals are housed individually, an increase in cortisol [40], depressive-anxious behaviors [44], and an increase in heart rate with an increase in sympathetic tone and a decrease in parasympathetic tone [8,41,45] have been observed. It has been hypothesized that exposure to prolonged psychosocial stress, such as the interruption of social connections, can affect key neuronal structures (e.g., decreased dendritic arborization in the hippocampus and increased dendritic arborization in the amygdala) involved in the regulation of behavioral, neurovisceral, and neuroendocrine responses [44,45]. Then, evidence from human studies in controlled settings such as those of space studies and or from the experience of COVID 19-related lockdowns has proved that if the activation of the response to social isolation is prolonged, it can lead to a chronic impairment of the homeostasis (Figure 1) and result in imbalanced cardiac activation and immune response, eventually leading to an increased risk of cardiovascular disease and immune dysregulation. Therefore, if this pattern of response was fundamental for the survival in the ancestral environment, nowadays it represents a risk for the health when chronically or prolongedly activated without real feedback of use.

In this regard, terrestrial spaceflight analogs and space missions are good models in which the effect of social isolation can be studied in a controlled way on a group of healthy subjects to understand what the target pathophysiological mechanisms are which underlie the relationship between social isolation, cardiovascular disease, and immune dysregulation and to allow the individuation of effective interventional countermeasures. 

## 3. Social Isolation and Cardiovascular Autonomic Control Alterations

Perceived loneliness and objective social isolation are two well-known independent cardiovascular risk factors [9,46,47]. In the literature, loneliness and social isolation have been correlated with increased total peripheral vascular resistance [15], the dysregulation of cardiovascular reactivity to stress [48], and an increased risk of developing hypertension [49], stroke, or coronary artery disease [50].

Social isolation may act on increased cardiovascular risk in two different ways. The first proposed mechanism is an “indirect effect” related to social behaviors conveyed by one’s social network (e.g., members of one’s social network would encourage people to eat a good diet or exercise and, conversely, discourage them from behaviors detrimental to physical health such as drinking or smoking [51]). The second hypothesis for the link between social isolation and cardiovascular risk involves the ANS. According to the neurovisceral integration model, there is a strong connection between the brain and the heart, mediated by the ANS [52]. The prefrontal cortex has a critical role in inhibiting the sympatho-excitatory subcortical threat circuits, in which the amygdala is involved [53]. In conditions perceived as threatening, there is a primary activation of the amygdala [54] and a hypoactivation of the critical areas of the prefrontal cortex that, among other effects, involve the acceleration of the heart frequency [53]. In the literature, loneliness scores have been negatively associated with regional white matter density of the prefrontal cortex [55]. Therefore, this model could offer a possible framework to explain mechanisms linking autonomic imbalance and cardiovascular risk [56]. 

In a physiological condition, the parasympathetic and sympathetic branches of the ANS operate in a dynamic balance at the cardiac level. As a result, a healthy heart does not have a regular inter-beat interval, but is regulated according to the stimuli present [57]. In contrast, an autonomic imbalance is related to a lack of flexibility, and therefore a compromised health status [58]. Therefore, the study of the cardiac sympatho-vagal balance could represent a valid source of information about the individual’s physical and mental health. 

Cardiovascular autonomic control can be investigated through heart rate variability (HRV) analysis, which considers the fluctuations in the temporal distance between consecutive beats. Indeed, from the analysis of the ECG and in particular from the measurement of the interval between beats, it is possible to derive indices that, properly analyzed, relate to sympathetic or parasympathetic modulation in the heart [59]. In particular, one of the main HRV analysis methods is spectral analysis, which allows the identification of two oscillatory components, namely low frequency (LF) and high frequency (HF). The HF band (ranging from 0.15 to 0.4 Hz) reflects parasympathetic activity, and its power is influenced by breathing, whereas the LF band (ranging from 0.04 to 0.15 Hz) seems to be produced by both sympathetic and parasympathetic branches, even if its physiological interpretation is still controversial [59]. Finally, the ratio of LF to HF power (LF/HF) provides information about the sympatho-vagal balance [60]. Alterations of ANS that promote vagal withdrawal are reflected in reductions of HRV. Studies on mental stress have shown that cardiac autonomic imbalance may play a central role in the association between social isolation, feelings of loneliness, and increased cardiovascular morbidity and mortality. Indeed, it is known that higher cardiac activity (heart rate) at rest and low HRV, indicative of PNS hypoactivity and SNS hyperactivity on cardiac control, are associated with increased cardiovascular morbidity and mortality [58,61,62,63]. Similar evidence was also derived from space studies. In particular, ground-based analogs have been built to overcome logistic, financial, and practical restrictions of space missions. Moreover, knowledge from studies on spaceflight analogs can permit the assessment of the effects of the single space hazards and develop innovative technologies aimed at reducing health risks in astronauts during spaceflight missions and in frail populations (e.g., old persons, hospitalized patients, depressed patients) [11]. Therefore, space analogs as controlled models allow the observation of the consequences of prolonged isolation on the human organism. 

In this perspective, ESA in 1990 undertook an experimental program centered on psychological problems that could afflict space crew to inquire into the role of confinement and isolation on physiological functions, without being spoilt by the influence of microgravity. They started with the “Isolation Study for the European Manned Space Infrastructure” (ISEMSI), which observed six men of different nationalities being isolated for 28 days (towards the end of the isolation period, two out of the six men were further isolated from the others) [64]. The second confinement study was the “Experimental Campaign for European Manned Space Infrastructure” (EXEMSI) in 1992, with three men and a woman being isolated for 60 days [65]. In 1994, the duration of the isolation experiment was prolonged to 135 days in the “Human Behavior Study” (HUBES), and up to 240 days in the “Simulation of the Flight of the International crew on Space Station” (SFINCSS) [66]. In 2009 the Mars105 project was carried out, followed in 2011 by the Mars500, exposing six crewmembers to 105 or 520 days of isolation and confinement, respectively; Mars520 turned out to be the longest isolation experiment in history [24,67]. ESA is now collaborating with NASA in a series of isolation studies as part of the “Scientific International Research in Unique Terrestrial Station” (SIRIUS) project. Meanwhile, NASA keeps simulating spaceflights in analog habitats, such as the NASA Extreme Environment Mission Operations (NEEMO), Hawai’i Space Exploration Analog and Simulation (HI-SEAS) and NASA’s Human Exploration Research Analog (HERA) [18]. 

The first observations about cardiovascular consequences of confinement and isolation came from the ISEMSI; at the beginning of the study, an increase in renin, aldosterone, and antidiuretic hormones levels as well as in systolic blood pressure (SBP) was reported [68], not only because of alterations in the hydro-electrolytes balance, but also due to confinement-related stress [69]. However, heart rate, HRV, and neuro-endocrine measures (i.e., catecholamines, cortisol and testosterone) did not show any alteration; it was then hypothesized that the relatively short duration of the isolation was sufficient only to slightly elevate stress levels.

In contrast to ISEMSI, during EXEMSI there was not a significant rise in SBP nor a change in the renin–angiotensin–aldosterone system (RAAS). This could be explained by considering that the activation of RAAS is interpreted as a defense reaction against a new situation and eventually against other subjects, but this response is mitigated when the new setting becomes familiar and when a moderating effect (in this case, the presence of a female crew member) is present [69]. On the other hand, tracking autonomic cardiovascular function showed that stress from confinement, even if not appreciably altering neural mechanisms underlying cardiorespiratory control [70], caused cumulative stress effects to emerge, in terms of higher cardiovascular load, in subjects carrying higher responsibilities during the mission, especially during the experimentation [71]. Depending upon confinement conditions, isolation seemed to have its effect at the beginning of the confinement period (EXEMSI) as well as during the entire confinement period (ISEMSI), and also varies according to each crew member’s personal response and role in the experimentation.

In Mars105, the HRV analysis verified a decreased mean heart rate and increased amplitude in all frequency components, along with a relative LF decrease compared to HF that resulted in a diminished LF/HF. It was also noticed that confinement resulted in an attenuation of the differences between the wake and sleep phases of the LF, HF, and LF/HF power. The relative decrease in the LF component seemed to show an augmented parasympathetic activity during the wake period, justifying the loss of sympathetic predominance in the wake period in addition to the vanishing of the sleep–wake HRV differences. These findings aligned with observations from a study involving nine male subjects that were isolated for a 40-day stay in the Italian Antarctic Station of Terra Nova Bay; it revealed an autonomic imbalance with a relative reduction in the SNS and/or a relative increase in the PNS, with a net prevalence of the latter, during both wake and sleep, that was possibly associated with a reduction in the pituitary–adrenal hormonal axis activity and a consequent decrease in adrenal hormonal levels [72]. 

This kind of HRV pattern during sleep and wake phases seems to be the result of a long-term confinement in which the percentage of sleep phases and rest rises, leading to a circadian misalignment that can determine a decreased performance during daytime tasks [18]. The disruption of circadian balance during isolation was further confirmed by Mars500 [73]. In this study, researchers found a progressive increase in the amplitude of the HF component during wake periods, while during sleep periods a decrease in HF was noticed; this corresponded to an augmented parasympathetic activity in daytime and a diminished parasympathetic activity during sleep periods, respectively. Hence, a loss of circadian HRV rhythms appeared to be induced by confinement, even over longer periods [73].

The dysregulation of circadian rhythm has been found also in depressive patients and has been proposed both as a consequence and a cause of depressive symptoms [74,75]. As a matter of fact, cardiovascular autonomic dysregulation was associated with sleep disorders and the insurgence of depressive-like behavior, such as anhedonia. Therefore, altogether these observations underlie a strict association between social isolation, autonomic circadian rhythm alteration and the onset of depressive symptoms.

The importance of HRV in influencing and investigating psychological health has also been observed during the COVID-19 lockdown period. Indeed, HRV is considered an effective method of measuring and regulating emotional response, as it is indicative of the flexibility with which the body is able to adapt to environmental changes. A high HRV value (indicative of predominant PNS control) is associated with greater flexibility in choosing the most appropriate behavioral response to the situation, thus becoming a protective factor in personal emotional regulation [52]. Being resilient to external stressors, such as social isolation, is useful during space missions or their terrestrial counterparts, just as it was during the period of isolation experienced during pandemic. Indeed, although not widely investigated in the literature, during the lockdown period it has been observed that those with higher HRV showed more functional emotion regulation strategies, and reported fewer depressive symptoms and greater subjective well-being [76,77]. 

Such studies underscore the importance of the ANS in mediating the relationship between the subject and the environment.

## 4. Social Isolation and Inflammation

Another hallmark of social isolation on human health is an enhanced pro-inflammatory state. In recent years, more and more studies have highlighted the link between behavior and inflammatory processes through a two-way relationship. However, if the involvement of a pro-inflammatory state in the etiopathogenesis of depressive symptoms such as social withdrawal and anhedonia (the so-called sickness behavior) is now increasingly investigated [78,79], the evidence of a direct action of social behavior on the inflammatory state is not as widespread. As a matter of fact, social isolation represents an important risk factor for the development of chronic diseases characterized by detrimental immune alterations (e.g., autoimmune diseases, type II diabetes, cancer), but also for an impairment in the response to viral infections [80]. The increased reactivation of latent herpes viruses including Epstein–Barr virus, varicella-zoster virus and cytomegalovirus was observed in more than half of the crew members in conjunction with increased plasma cytokine levels [81]. The reactivation and shedding of latent viruses and an accompanying reduction in cell-mediated immunity were also observed in ground-based space analogs during the Antarctic winter, when social isolation is at its maximum due to extreme environmental conditions [82].

Once again, spaceflights and space analogs are congenial experimental contexts for studying the effects of social isolation on a pro-inflammatory state, and therefore immune function. The extreme conditions related to the space environment influence biological features, mainly inducing changes in molecular and cellular mechanisms associated with the adaptation to stress. Indeed, mechanisms related to stress response, such as DNA damage, oxidative stress, mitochondrial dysregulation, epigenetic changes, and telomere shortening, have been extensively described in relation to spaceflight [83]. Among the stressors to which astronauts are subjected, social isolation has increasingly emerged as having a fundamental role in the above-mentioned alterations. 

As a matter of fact, social isolation and confinement during long-duration spaceflight were observed to determine a significant increase in the plasma and salivary levels of several cytokines with respect to pre-mission conditions, determining a persistent pro-inflammatory state [81,84]. Furthermore, severe impairments of immunological function were associated with latent viral reactivation [81]. The altered distribution of peripheral leukocytes, the diminished function of specific leukocyte subpopulations and skewed cytokine profiles have also been described in many astronauts [20,22,85]. Interestingly, an increase in IL-8 and TNFa levels were associated with a low-grade inflammatory status during spaceflight [22,86], similar to those experienced in old persons [87]. The plasma levels of IL-1ra, an inhibitor of the proinflammatory IL-1, were also consistently elevated during spaceflight [86], probably due to an attempt at an adaptive physiological response to stress [88]. Similar alterations to the inflammatory profile were also found in crew members of Antarctica winter-over and 1-year missions at the French–Italian Concordia station and Australian National Antarctic Research Expeditions stations [82,85]. Moreover, T-cells were significantly decreased in number and activity, whereas monocytes and granulocytes were increased, as well as the oxidative activity of granulocytes and the consumption of anti-oxidative resources [89,90]. The monitoring of immune function during 14-day missions conducted in the underwater deployment NASA Extreme Environment Mission Operations (NEEMO) revealed an increase in cytokines production during the later phases of the stay [22]. The immune changes identified during the longest isolation study, Mars520, were a significant increase in lymphocytes and a heightened immune response [91]. 

The mechanisms underlying the establishment of these immune changes and latent viral reactivation seem to be the result of an evolutionary process that selected them as adaptative responses to social isolation. Social connection has always been a key factor for human survival and, on the other hand, social isolation and confinement appear to be stressors as they jeopardize the safety of the subject, exposing the individual to predators and increasing the risk of wounds and infections. 

Thus, the environment determines a specific stress response for a possible future scenario characterized by the risk of wound infection, but also by a lower viral exposure given the social disconnection. The up-regulation of inflammatory activity and down-regulation of antiviral responses observed from space studies could be interpreted as the result of an adaptive response to social isolation conceptualized by Cole and colleagues and termed as the conserved transcriptional response to adversity (CTRA), in which, once again, the ANS acts as the principal mediator between the stress factor and the body adaptation [92,93,94]. In particular, the SNS activates the CTRA in response to chronic social isolation through β-adrenergic receptors, resulting in the multiple control of different transcription pathways towards the up-regulation of pro-inflammatory gene transcription and the down-regulation of Type I interferon-mediated innate antiviral responses [95]. It is of interest to note that in old persons, isolation and confinement also play a crucial role in deviating the trajectories of aging from a good trajectory (longevity) to a bad one (burden by diseases) [96], with high levels of loneliness associated with increased proinflammatory response and altered antiviral gene expression, which resumes the CTRA profile [94].

The extent of the duration of the stressful event, as well as the recruitment of the CTRA, is of primary importance in the shift from adaptive condition to dysfunctional state, with the consequent establishment of a low-grade chronic inflammation. When an acute stress occurs, the physiological response is short-lasting and ceases immediately upon removal of the stressor. In the case of chronic stress or persistent stressful events, the prolonged recruitment of the same response to acute stress can result in immune dysregulation or suppression, as shown by data from spaceflights and space analogs [22,85]. 

The link between psychosocial stressors and the activation of the pro-inflammatory response has been also observed during the COVID-19 lockdown. A study using brain imaging techniques (simultaneous positron emission tomography and magnetic resonance spectroscopy) found, in a group of uninfected patients, elevated levels of two independent neuroinflammatory markers (18 kDa translocator protein—TSPO, and myoinositol) and elevated, although non-significant, levels of systemic inflammatory markers (IL-16 and monocyte chemoattractant protein-1, IL-6 and MCP-1) in the post restrictive measures condition compared with the pre-pandemic period [97]. The variables of mental/physical fatigue, difficulties in cognition, and of mood alterations were also analyzed, finding a correlation between higher burden symptoms and a higher TSPO signal in the brain. In addition, this finding has been linked to higher levels of IL-6 and MCP-1 [97]. 

Another study found a significantly increased C-reactive protein (CRT) in subjects undergoing a routine hematologic examination in the post-lockdown condition compared to the pre-pandemic condition, and the authors linked this evidence of increased mild systemic inflammation to the stress experienced due to isolation and physical inactivity [98]. However, in this latest paper, the authors did not mention whether or not the analyzed sample was negative for COVID-19 infection, so there may be a bias in the enrolment.

This finding highlights the importance of the bidirectional relationship between psychosocial stress and inflammation: a chronic condition of psychosocial stress upregulates the pro-inflammatory response, which in turn has been linked to the presence of so-called sickness behavior and the presence of depressive symptomatology. Therefore, it is of great importance to consider the presence of this relationship and take action to anticipate the formation of this vicious cycle. 

## 5. Conclusions and Future Perspectives

The human being has always had gregarious behaviors motivated by an evolutionary drive advantageous for survival. Even today, social isolation and confinement are particularly stressful factors and are used as forms of torture or punishment, as happens in the case of imprisonment.

The studies conducted in the context of space missions and space analogs have a strong value that should not be neglected in specific frail populations or in public health, as seen during the COVID-19 lockdown periods. As a matter of fact, the possibility of studying the mechanisms that underlie the consequent pathophysiological alterations caused by prolonged social isolation in controlled environments can help to reason around possible countermeasures and verify their effectiveness.

Moreover, considering the plans for the extension of space missions or the colonization of Mars, it is important to consider psychosocial factors in addition to the physical stressors present during missions (e.g., microgravity or circadian cycle misalignment). Among these, social isolation has a major role, not only because of its impact on mental health and cognitive capacity, but also for the repercussions observed on physical health. Indeed, the physiological response triggered by social isolation is adaptive in the short term, but it also requires a rapid return to the initial state of equilibrium in order not to have the detrimental effects of prolonged activation.

As emerged from our review, the ANS could represent an interesting target to counteract the negative effects of stress response when the removal of the stressful stimulus is not possible, as it represents the main mediator of internal regulation with respect to external stimuli. Notably, there are non-pharmacological methods, such as transcutaneous auricular vagus nerve stimulation (taVNS), that are cheap and non-invasive ways to effectively act on the autonomic imbalance. For example, the stimulation of the parasympathetic branch of the ANS has already proved to be effective in remodulating cardiovascular autonomic control, thus reducing the risk of developing adverse cardiovascular alterations, and in reducing even low-grade chronic inflammation states. Therefore, future perspectives should address the application and study of this new countermeasure in good study models of social isolation (such as spaceflights and space analogs) and in cohorts of frail subjects exposed to social isolation.

## Data Availability

Not applicable.
